# Functional Correlates of Microglial and Astrocytic Activity in Symptomatic Sporadic Alzheimer’s Disease: A CSF/^18^F-FDG-PET Study

**DOI:** 10.3390/biomedicines11030725

**Published:** 2023-02-28

**Authors:** Chiara Giuseppina Bonomi, Agostino Chiaravalloti, Riccardo Camedda, Francesco Ricci, Nicola Biagio Mercuri, Orazio Schillaci, Giacomo Koch, Alessandro Martorana, Caterina Motta

**Affiliations:** 1 UOSD Memory Clinic, Policlinico Tor Vergata, University of Rome “Tor Vergata”, 00133 Rome, Italy; 2 Department of Biomedicine and Prevention, University of Rome “Tor Vergata”, 00133 Rome, Italy; 3 IRCCS Neuromed, 86077 Pozzilli, Italy; 4 Non-Invasive Brain Stimulation Unit, IRCCS Santa Lucia, 00179 Rome, Italy; 5 Human Physiology Unit, Department of Neuroscience and Rehabilitation, University of Ferrara, 44121 Ferrara, Italy

**Keywords:** astrocytes, FDG-PET, Alzheimer’s Disease, biomarkers, CSF

## Abstract

Glial and microglial cells contribute to brain glucose consumption and could actively participate in shaping patterns of brain hypometabolism. Here, we aimed to investigate the association between ^18^F-fluorodeoxyglucose (^18^F-FDG) uptake and markers of microglial and astrocytic activity in a cohort of patients with Alzheimer’s Disease (AD). We dosed cerebrospinal fluid (CSF) levels of soluble Triggering Receptor Expressed on Myeloid cells (sTREM2), Glial Fibrillary Acidic Protein (GFAP), a marker of reactive astrogliosis, and β-S100, a calcium-binding protein associated with a neurotoxic astrocytic profile. No associations were found between sTREM-2 and 18F-FDG uptake. Instead, ^18^F-FDG uptake was associated negatively with CSF β-S100 in the left supramarginal gyrus, inferior parietal lobe and middle temporal gyrus (Brodmann Areas (BA) 21 and 40). Increased β-S100 levels could negatively regulate neuronal activity in the temporo-parietal cortex to prevent damage associated with AD hyperactivity, or rather they could reflect neurotoxic astrocytic activation contributing to AD progression in key strategic areas. We also identified a trend of positive association of ^18^F-FDG uptake with CSF GFAP in the right fronto-medial and precentral gyri (BA 6, 9 and 11), which has been reported in early AD and could either be persisting as an epiphenomenon tied to disease progression or be specifically aimed at preserving functions in the frontal cortex. Overall, CSF markers of astrogliosis seem to correlate with cortical glucose uptake in symptomatic sporadic AD, highlighting the role of astrocytes in shaping regional hypometabolism and possibly clinical presentation.

## 1. Introduction

Alzheimer’s Disease (AD) is marked by the accumulation of amyloid-β (Aβ) peptides, which according to the amyloid hypothesis triggers tau-hyperphosphorylation and its intraneuronal deposition [1]. Despite these two pathological changes being the main actors of AD, it is widely recognized that many transversal mechanisms participate in its pathophysiology, acting alongside Aβ and tau pathology [2]. For instance, AD is often complicated by comorbidities, including diabetes and other metabolic conditions, which further increase the complexity of disease mechanisms [3,4].

Specifically, unravelling the interplay between cellular neuroinflammatory contributions—i.e., microglial activation and astrocytic reactivity—amyloid pathology and tauopathy has been the object of many efforts [5,6,7], because of their interesting and possibly ambivalent role in the progression of the disease.

Microglia regulates neuronal proliferation and the phagocytic removal of apoptotic neurons [8], de-facto shaping brain circuitry/connectivity, and its alterations play a key and intricate role in AD [9]. Indeed, loss-of-function mutations of Triggering Receptor Expressed on Myeloid Cells 2 (*TREM-2*)—a gene encoding a transmembrane receptor expressed on brain microglia—are the second genetic risk factor for sporadic AD, after Apolipoprotein E (*APOE*) [10]. The TREM-2 cascade has been discussed for its seemingly dual role since, on the one hand, it favors the phagocytic removal of Aβ while on the other it could mediate and sustain chronic damaging effects [11,12]. Similarly, reactive astrocytosis, marked by changes of astrocytic morphology within and around neuritic plaques, is also a typical finding in AD, despite whether its role is protective or detrimental still being vastly debated [13,14].

These cellular mechanisms have been much explored through their cerebrospinal fluid (CSF) correlates. Indeed, an increase of the soluble form of the TREM-2 receptor (sTREM-2) has been thoroughly reported from the early phases to more advanced stages of AD [15,16,17,18] and linked with reduced rates of amyloid accumulation in several studies [16,19], configuring it as a reliable marker of AD-related microglial activation. Many studies have also reported changes in the astrocytic secretome profile occurring in response to AD pathophysiology [20,21], including, among others, the increased production of both plasma and CSF levels of Glial Fibrillary Acidic Protein (GFAP)—the main intermediate filament protein in mature astrocytes—[22,23] and β-S100, a calcium-binding protein tied to neurotoxic astrocytic activity [24,25]. In a recent work, we ourselves investigated these CSF correlates of microglial and astrocytic activation, namely CSF sTREM-2, GFAP and β-S100, with our results supporting a dynamic switch in microglial functions, from neuroprotective to neurotoxic, depending on disease stage and APOE genotype, and a tight association of microglial activity with astrocytic reactivity, and with the acquisition of a more neurotoxic astrocytic phenotype (in this study).

The importance of the glial compartment has also been validated by recent studies highlighting the contribution of astrocytic metabolism to ^18^F-fluorodeoxyglucose (^18^F-FDG) uptake during Positron Emission Tomography (PET) scans [26,27]. Strong evidence supports the hypothesis that glutamate recycling in astrocytes activates aerobic glycolysis, with neurons being partially fueled by lactate derived from astrocytes [28], and astroglial glutamate transport triggering glucose uptake by astrocytes [29]. On the other hand, microglial cells have been shown to consume more glucose than neurons and astrocytes, and their activation state has likewise been linked to FDG-PET alteration in AD mouse models and AD patients [30]. This leads to speculation that microglial and astrocytic activity might shape patterns of regional hypometabolism of ^18^F-FDG-PET and, hence, cognitive manifestations of AD, proving the relevance of investigating the associations between astrogliosis, microgliosis and cortical metabolism.

Previous works also investigated the relationship between measures of cerebral glucose consumption and some markers of neuroinflammation, focusing especially on pre-symptomatic or very early AD, highlighting the presence of higher ^18^F-FDG-PET uptake in relation to neuroinflammatory processes in early AD [31,32,33]. We aimed to implement these findings by evaluating a cohort of symptomatic patients diagnosed with sporadic AD, by exploring possible correlations between regional hypometabolism of ^18^F-FDG-PET and changes in CSF biomarkers of microglial and astrocytic reactivity (sTREM-2, GFAP and β-S100).

## 2. Materials and Methods

### 2.1. Subjects’ Enrolment

Between September 2021 and December 2021, we enrolled 35 outpatients that had been referred to the UOSD Centro Demenze of the University Hospital “Policlinico Tor Vergata” in Rome upon suspicion of Alzheimer’s Disease (AD). After initial assessment, all patients underwent a complete diagnostic work-up including Mini Mental State Examination (MMSE), corrected by age and education, laboratory testing to rule out secondary cognitive decline, 3T brain MRI, and lumbar puncture. All patients also underwent ^18^F-FDG-PET at the Nuclear Medicine Unit of University Hospital “Tor Vergata”.

Eventually, 31 patients received a CSF biomarker-based diagnosis of AD according to the most recent NIA-AA research framework (i.e., as having an increase of CSF p-tau alongside Aβ42 decrease, A+T+, or sole evidence of amyloid pathology, A+T−). Genetic testing for Apolipoprotein E (APOE) was also performed on all subjects. All patients showed neuropsychological profiles compatible with classical AD [34].

We excluded patients ongoing treatment with antipsychotic drugs or having major comorbidities, such as oncological history, inflammatory/autoimmune systemic conditions, decompensated diabetes, or organ failure. Other exclusion criteria were a history of manifest acute stroke (i.e., Hachinski scale score > 4 or radiological evidence of focal ischemic lesions) and clinical evidence or suspicion of other neurological disorders. Thus, our final sample included 27 patients within the Alzheimer’s continuum (ADc) [35], namely 6 A+T− and 21 A+T+. All subjects included were right-handed. Demographics from patients are reported below (see Table 1).

We obtained written consent from all participants and/or legally authorized representatives. The ethical committee of the Santa Lucia Foundation accounted for the study protocol as an observational retrospective design (Prot. CE/AG4/PROG.392–08).

### 2.2. CSF Sampling and Laboratory Analysis

All lumbar punctures were performed with sterile technique between 8 and 10 am. A 10 mL CSF sample was collected for each patient in polypropylene tubes. A total of 2 mL was used for routine biochemical analysis and we centrifuged the remaining 8 mL at 2000× *g* at +4 °C for 10 min and aliquoted them in 1 mL portions. The aliquots were frozen at −80 °C for further analysis of CSF, AD, and glial biomarkers. We used commercially available kits for biochemical analysis. CSF Aβ42, p-tau and t-tau concentrations were determined using a sandwich enzyme-linked immunosorbent assay (EUROIMMUN ELISA©), LUMINEX© Multiple assays ELISA was used for CSF GFAP and β-S100 concentrations.

Blood samples were also drawn in EDTA tubes. The DNA was extracted automatically and APOE genotyping was conducted by allelic discrimination technology with real-time PCR, according to the manufacturer’s instructions (TaqMan; Applied Biosystems).

### 2.3. ^18^F-FDG-PET Data Acquisition

All acquisitions were performed at the Nuclear Medicine Unit of the University Hospital “Policlinico Tor Vergata” in Rome with a General Electric VCT PET/CT scanner (GE Medical Systems, Tennessee, USA). All subjects fasted for at least 5 h before i.v. injection of FDG, and serum glucose levels were in range according to European Association of Nuclear Medicine guidelines [36]. Patients were injected intravenously with ^18^F-FDG (dose range 185–295 MBq) and then hydrated with 500 mL of saline (0.9% sodium chloride), according to a previous similar report of our group in this field [37]. The scan started 30 min after the injection and lasted ten minutes. Acquisition and reconstruction parameters are reported elsewhere and were followed according to the report cited above. [37].

Upon imaging evaluation, all 27 subjects showed typical findings of brain cortical hypometabolism in key regions compatible with a diagnosis of classical AD (parietal and posterior cingulate cortices, precuneus, or a combination of the above) [38].

### 2.4. Statistical Analysis

The relationship between levels of CSF GFAP, sTREM-2 and βS100 biomarkers and brain ^18^F-FDG uptake were analyzed through separate correlation models for each biomarker and direction, performed using Statistical parametric mapping (SPM) 12 (Wellcome Department of Cognitive Neurology, London, UK; https://www.fil.ion.ucl-.ac.uk/spm/software/spm12/, accessed on 1 January 2023) implemented in Matlab 2018 (Mathworks, Natick, MA, USA).

PET data were converted from DICOM to Nifti format using the MRIcron software (available at https://www.nitrc.org/projects/mricron/, accessed on 1 January 2023) and then subjected to normalization. Bias regularization was applied (0.0001) to limit distortions due to smooth, spatially varying artifacts that can modulate image intensity and interfere with automated image processing. The FWHM of Gaussian smoothness of distortion (to prevent the algorithm from trying to model out intensity variations due to different tissue types) was set to a limit of 60 mm; the tissue probability map implemented in SPM12 was used (TPM.nii). An affine registration with mutual information with the tissue probability maps [39] was used to achieve approximate alignment with the ICBM spatial template—European brains [40,41]. Warping regularization was set with the following 1 × 5 arrays (0, 0.001, 0.5, 0.05, 0.2); smoothing (to cope with functional anatomical variability not compensated by spatial normalization and to improve signal-to-noise ratio) was set to 5 mm; sampling distance (encoding the approximate distance between sampled points in estimating model parameters) was set to 3.

We used an 8 mm isotropic Gaussian filter to blur individual variations (especially gyral variations) and increase the signal-to-noise ratio. Prior to regression analysis, the following parameters and post-processing tools were used: global normalization (which brings the images to a global value) = 50 (using proportional scaling); masking threshold (which helps identify voxels with acceptable signal in them) was set to 0.8; transformation tool of statistical parametric maps to normal distribution; correction of SPM coordinates to match Talairach coordinates, subroutine implemented by Matthew Brett (http://www.mrc-cbu.cam.ac.uk/Imaging, accessed on 1 January 2023). Brodmann surfaces (BA) were determined within a range of 0 to 3 mm from the corrected Talairach coordinates of the SPM output isocenter using a Talairach client (available at http://www.talairach.org/index.html, accessed on 1 January 2023). As suggested by Bennett et al. [42], sPM t-maps were calculated for multiple comparisons using the false discovery (*p* < 0.05) and corrected for multiple comparisons at the cluster level (*p* < 0.001).

The level of significance was set at 100 (5 × 5 × 5 voxels, i.e., 11 × 11 × 11 mm) contiguous voxels. The voxel-based analyses were performed in the subgroup of patients using regression analyses. We designed independent models assessing the effects of each CSF biomarker (levels of GFAP, sTREM-2 and βS100) as regression factors—independent variables—on cortical ^18^F-FDG uptake, using age and sex as covariates, as well as t-tau levels in order to adjust for another marker of neurodegeneration. Positive and negative associations were tested in all cases.

The cluster obtained in the regression analysis was then exported by means of the WFU Pickatlas tool implemented in SPM 12. Specifically, the mean signal intensities calculated from each cluster within each subject were normalized to the average intensities of the Pons volume of interest. The use of normalization based on activity in the pons, rather than whole-brain counts as in the reference region, has been reported to result in greater accuracy in discriminating patients from controls in neurodegenerative diseases. As previously suggested by Pagani and colleagues [43]), a dataset of normalized ^18^F-FDG values relevant to the cluster under study was exported. To determine whether the normalized ^18^F-FDG values for the studied cluster were Gaussian distributed, the D’Agostino K-squared normality test was applied (with the null hypothesis being normal distribution). We then performed linear univariate regression analyses using GraphPad Prism© version 9.3.1 for Windows (GraphPad Software, San Diego, California USA, www.graphpad.com, accessed on 1 January 2023) to visualize significant findings, namely the association between CSF glial biomarkers and normalized continuous data representing metabolism in the specific Broadman Areas of interest.

## 3. Results

**Figure 1:**
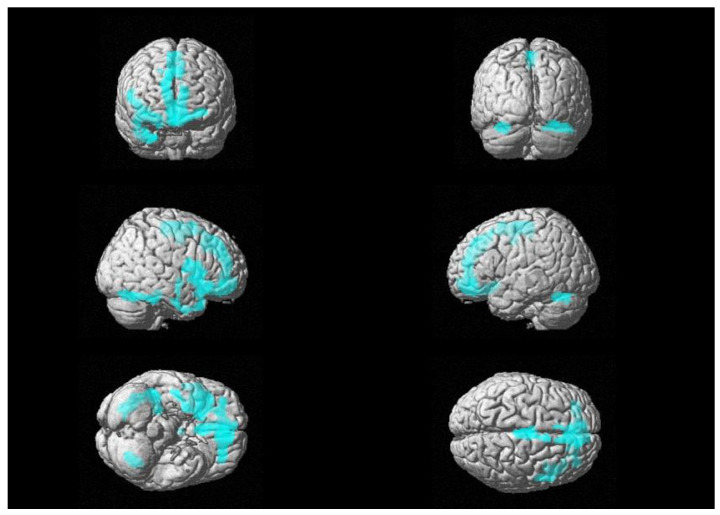
Three-dimensional brain rendering showing clusters obtained in SPM regression analysis for the positive correlation between CSF GFAP and glucose ^18^F-FDG uptake (see Table 2).

First, we retrieved a trend of positive association between ^18^F-FDG uptake and CSF levels of GFAP in the right frontal-medial and precentral gyri (see Figure 1), with peaks in BA 6, 9 and 11 [see Table 2].

**Figure 2:**
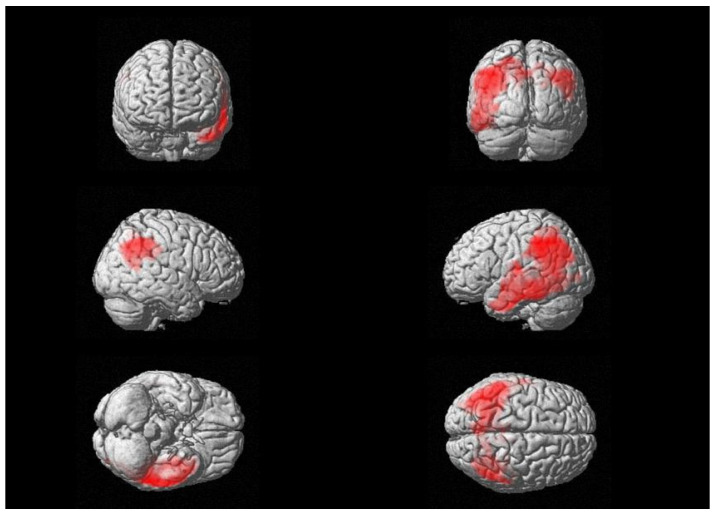
Three-dimensional brain rendering showing clusters obtained in SPM regression analysis for the negative correlation between CSF β-S100 and brain ^18^F-FDG uptake (see Table 3).

Moreover, a significant negative relationship between brain glucose consumption and CSF levels of β-S100 was also found in a wide cluster that included the left supramarginal gyrus, the inferior parietal lobe and middle temporal gyrus (see Figure 2), with peaks in Brodmann Areas 21 and 40 [see Table 3]. We did not find any significant relationships between ^18^F-FDG uptake and CSF levels of sTREM-2.

Lastly, we performed linear regression analyses to assess the association between glial CSF biomarkers and metabolism in the specific Broadman Areas of interest.

**Figure 3:**
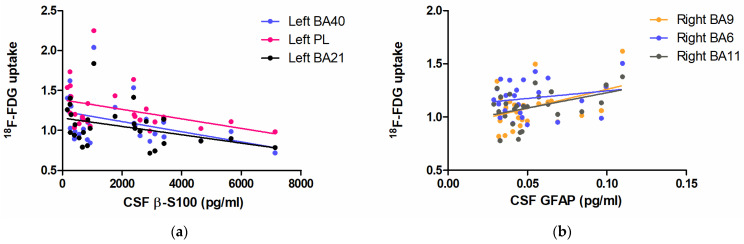
Scatter plots demonstrating the linear relationship between CSF β-S100 and ^18^F-FDG brain uptake in the left BA40 (blue), left BA21 (black), and the enlarged hub in the homolateral parietal lobe (PL, pink) (**a**); the relationship between CSF GFAP and ^18^F-FDG brain uptake in the right BA9 (orange), BA6 (blue) and BA11 (grey) (**b**).

Figure 3a shows regressions between β-S100 and ^18^F-FDG brain uptake in BA40 (R2 = 0.1593, F (1,27) = 4.737, *p* = 0.0392), BA21 (R2 = 0.1440, F (1,27) = 4.207, *p* = 0.0509) and the widened cluster of the parietal lobe (R2 = 0.1460, F (1,27) = 4.275, *p* = *0*.0492).

Figure 3b shows the linear regression between CSF GFAP and ^18^F-FDG brain uptake in BA9 (R2 = 0.1806, F (1,27) = 5.069, *p* = 0.0342), BA11 (R2 = 0.1705, F (1,27) = 4.727, *p* = 0.0402) and BA6 (R2 = 0.0313, F (1,27) = 0.9420, *p* = 0.3419).

## 4. Discussion

Despite its widespread use in both clinical settings and basic research, the identity of the cell types contributing to ^18^F-FDG-PET signal is still debated. Traditionally, ^18^F-FDG-PET signal has been attributed only to neuronal uptake, with hypometabolism being considered as a direct index of neuronal dysfunction—loss of neuropil, synapse, or functional impairment—or death. However, evaluating glucose consumption in AD is made complex by other additional factors, including changes in the expression of glucose transporters (GLUT) (e.g., a reduction of GLUT-3), insulin/insulin-growth-factor 1 axis dysregulation and defects due to the disruption of the neurovascular unit [44,45,46]. Moreover, other metabolically active cells are thought to contribute to glucose consumption in the brain [26,30].

Firstly, we observed a strong negative association between levels of CSF β-S100 and ^18^F-FDG cortical uptake in a wide cluster including the left supramarginal gyrus, the parietal lobe, and the middle temporal gyrus, with peaks in Brodmann Areas 21 and 40. To our knowledge this association is a novel finding, since relationships between this component, CSF β-S100, and cortical metabolism have never been described in symptomatic AD. Of note, one previous work reported no association between these two variables in a group of around 89 subjects (of which 25 A+T+) from the ALFA+ cohort, which includes pre-symptomatic and very early symptomatic patients with AD [31]. Thus, this result seems to be tightly bound to disease mechanisms that apply to late stages of the disease, after the onset of cognitive decline.

As a calcium binding protein, CSF β-S100 plays an active role in excitatory neurotransmission, as its exogenous form increases calcium concentrations in both cultured neurons and astrocytes [47]. By affecting intracellular Ca2+-dependent processes, extracellular β-S100 modulates synaptic plasticity, especially long term potentiation [48], and is linked to both hippocampal and non-hippocampal cognitive symptoms of AD [49].

Extensive data confirms that AD is marked by the presence of excitatory/inhibitory imbalance supported by Aβ-induced hyperexcitability [50,51] and built on pyramidal neuron hyperexcitability—that is also unsupervised due to GABAergic dysfunction [52]—and inhibition of glutamate reuptake [53]. Indeed, both AD-derived astrocytes and neurons cultured with AD astrocyte conditioned medium show aberrant intracellular Ca2+ dynamics in response to glutamate [54]. In light of this, speculations can be made that the increase of CSF β-S100 could reflect an attempt to negatively regulate regional neuronal activity, with the beneficial effect of preventing the neuronal damage associated with hyperactivity, which would result in decreased cortical metabolism and reduced ^18^F-FDG uptake. Alternatively, increased β-S100 could account for the local presence of pro-apoptotic neurotoxic astrocytes, favoring disease progression in areas that are typically involved in AD. In either scenario, our findings seem to point to the presence of increased regional vulnerability and/or incipient local neurodegeneration tied to astrocytic activation, with the increase of β-S100 acting either as a partial buffer or inducing neurotoxicity.

Moreover, since the association has been retrieved in key strategic areas of the dominant hemisphere, such as left BA40 and BA21, it could have repercussions on clinical presentation. Both areas belong to linguistic hubs, and are involved with language comprehension, semantic processing, and sentence generation (BA21, middle temporal gyrus) and with phonological abilities and verbal creativity (BA40, inferior parietal lobe, supramarginal gyrus) [55]. BA40 also plays a key role in several other functions, among which are working memory, motor functions (executive control of behavior and imitation, visuomotor transformation/motor planning), deductive reasoning, social perception and empathy [56]. This implies the need for a better understanding of the relationship between CSF levels of β-S100 and cognitive profile, to explore its possible role as a specific biomarker of these dysfunctions. Indeed, subtle impairment in both comprehension and strategic thinking, as well as all other cited cognitive domains, are often underrecognized in AD. Moreover, the identification of specific regional hypometabolic changes/hypoactivity suggests that targeted therapeutic approaches, such as the restoring of excitatory/inhibitory balance, could be beneficial to these symptoms.

Our second finding was the trend of positive association between CSF GFAP levels and glucose consumption in the right medial frontal gyrus, with maximum z-score of correlation peaking in BAs 9, 6 and 11. Since astrocytic reactivity is characterized by both morphological changes—resulting in cellular hypertrophy—and upregulation of GFAP expression and production, our finding supports the presence of active astrogliosis and higher metabolic demands in those areas of the frontal lobes. Previous studies focused on early AD, reported the presence of the same positive association between CSF GFAP and regional metabolism in both the frontal and the temporo-parietal lobes [32]. The loss of this linear association in the temporo-parietal and left frontal regions, which are among the first hubs to be damaged in AD [57], could be due to disease progression in our cohort of patients with symptomatic AD. Conversely, the presence of GFAP-related astrogliosis in the frontal lobe could either be an epiphenomenon resulting from astrocytic dysfunction in other areas—incapable of sustaining higher metabolic demands—or rather be specifically aimed at preserving functions in the frontal cortex in the course of symptomatic AD, supporting the hypothesis of its delayed involvement.

Interestingly, a previous work reported that higher CSF t-tau levels correlate with hypometabolism in the right frontal cortex [58], and the concordance between these and our findings suggests that the frontal lobe is a hot spot for the progression of AD. Astrocytic reactivity might sustain but also be exacerbated by tau/neurodegenerative changes in the frontal lobe, with increased GFAP production and higher metabolic demands possibly trying to sustain damaged circuitry.

In a work from Salvadó et al. (2022) [32], the positive GFAP/glucose consumption association turned negative when switching from isolated amyloidopathy (A+T-) to patients with full-blown AD (hence, with tauopathy: A+T+), which they interpreted as an uncoupling of astrogliosis from metabolism due to failure to sustain elevated energetic demands when tauopathy sets in. Our results suggest that the positive association between CSF GFAP and glucose uptake could uncouple in regions with higher damage but could also be partially maintained in the frontal lobes in symptomatic AD, even in the presence of tau pathology. In light of this, it would be very interesting to explore stage-dependent glucose metabolic changes associated with reactive astrogliosis separating symptomatic A+T- from A+T+, to solve this discrepancy.

Finally, in our cohort, we did not retrieve any significant correlation between CSF sTREM-2 and cortical glucose uptake. This finding contrasts with previous literature showing a link between sTREM-2 and FDG-PET signal in AD patients with Mild Cognitive Impairment (MCI). Biel and colleagues reported that sTREM-2 positively associates with FDG-PET hypermetabolism in patients with CSF findings of amyloidopathy but negative amyloid-PET, while, in case of amyloid-PET positivity—reflecting higher levels of fibrillary Aβ—sTREM-2 associates with hypometabolism instead [33]. Nevertheless, this discrepancy could be due to our limited sample size and to the different clinical and AT profiles of our cohort.

We are aware that this study has limitations. First, our study design is observational and cross-sectional, therefore no assumptions on direct causal relationships can be made, and it is not possible to completely decipher the mechanisms underlying these associations. Also, widening the study cohort could be useful to confirm and to strengthen our results, by repeating the assessments in A+T- and A+T+ patients. In addition, a larger sample size would allow the assessment of direct repercussions on specific cognitive domains.

Nonetheless, our results open several interesting future directions. The difference between our own findings and previous literature on cognitively unimpaired patients with AD stimulates the urge for follow-up longitudinal data, to add meaningful information on the burden of glial and microglial contributions on disease progression. Since it seems to be more sensitive to amyloid changes and have widespread correlation with cortical hypometabolism in early AD [32], it would also be interesting to use plasma GFAP instead of its CSF counterpart. Lastly, implementing the use of novel PET radiotracers such as TSPO for microglial activation could add more information on the bond between microglial inflammation and reactive astrogliosis.

## 5. Conclusions

Our results suggest that astrogliosis (CSF GFAP) in the frontal lobe is associated with higher local metabolic demands, while a negative association of CSF β-S100 with cortical metabolism was found in the parieto-temporal lobe, likely reflecting regional vulnerability to incipient damage. No microglial involvement with cortical glucose uptake has been identified in our study.

Overall, these findings represent glucose metabolic changes associated with reactive astrogliosis, adding evidence to the role of astrocytes in shaping ^18^F-FDG-PET signal in vivo. This also fuels the need to implement measures of glial and microglial activity in the stratification and profiling of patients, to allow a tailored understanding of pathophysiology and to better account for their contribution in the biological evolution of AD.

## Figures and Tables

**Table 1:** Demographic data and CSF analysis results expressed as means ± standard deviations.

	Study Cohort (n = 27)
Age (years)	67.52 ± 6.74
Sex (M:F)	13:14
MMSE	24.42 ± 3.74
APOE (E3:E4)	16:11
CSF Aβ42 (pg/mL)	390.05 ± 73.77
CSF p-tau (pg/mL)	98.48 ± 49.13
CSF t-tau (pg/mL)	585.22 ± 369.92
CSF sTREM-2 (pg/mL)	1.47 ± 0.25
CSF GFAP (pg/mL)	0.05 ± 0.02
CSF β-S100 (pg/mL)	1936.41 ± 1807.22

CSF, cerebrospinal; MMSE, Mini-Mental State Examination; APOE, Apolipoprotein E; sTREM2, soluble Triggering Receptor Expressed on Myeloid Cells 2; GFAP, Glial Fibrillary Acidic Protein.

**Table 2:** Multiple regression analysis showing the CSF GFAP related areas of increased ^18^F-FDG brain uptake (positive correlation), corrected for age, sex and CSF levels of t-tau.

Analysis	Cluster Level					Voxel Level		
Positive correlation (GFAP)	**Cluster p (FEW-corr)**	**Cluster p** **(FDR-corr)**	**Cluster extent**	**p** **unc**	**Cortical region**	**z-score of maximum**	**Talairach** **coordinates**	**Cortical region**
	0.533	0.446	2943	0.023 *	R Medial Frontal Gyrus R Precentral Gyrus R Medial Frontal Gyrus	3.46 3.08 2.92	12 42 26 8 32 36 −6 42 −8	BA9 BA6 BA11

In the Cluster level section (left), the number of voxels, the uncorrected and corrected *p*-value of significance and the cortical region where the voxel is found are reported for each significant cluster. In the Voxel level section (right), all coordinates of the correlation sites (with z-score of the maximum correlation point) and the corresponding subcortical region are reported for each significant cluster. L: left; R: right; FEW: Familywise error; FDR: False discovery rate; BA: Broadman Area; p unc: uncorrected p, * = *p* < 0.05.

**Table 3:** Multiple regression analysis showing the CSF β-S100 related areas of decreased ^18^F-FDG brain uptake (negative correlation), corrected for age, sex and CSF levels of t-tau.

Analysis	Cluster Level				Voxel Level		
Negative correlation (β-S100)	**Cluster p (FEW-corr)**	**Cluster p** **(FDR-corr)**	**Cluster extent**	**Cortical region**	**z-score of maximum**	**Talairach** **coordinates**	**Cortical region**
	0.001 ***	0.000 ***	13,253	L Supramarginal Gyrus L Middle Temporal Gyrus L Inferior Parietal Lobe	3.49 3.25 3.20	−42 −54 46 −58 −26 −12 −50 −40 42	L BA40 L BA21 L BA40

In the Cluster level section (left), the number of voxels, the corrected *p*-value of significance and the cortical region where the voxel is found are all reported for each significant cluster. In the Voxel level section (right), all coordinates of the correlation sites (with z-score of the maximum correlation point) and the corresponding subcortical region are reported for each significant cluster. L: left; R: right; FEW: Familywise error; FDR: False discovery rate; BA: Broadman Area, *** = *p* ≤ 0.001.

## Data Availability

Data are available upon reasonable request.

## References

[1] Bloom G.S. (2014). Amyloid-β and Tau: The Trigger and Bullet in Alzheimer Disease Pathogenesis. JAMA Neurol..

[2] Guo T., Zhang D., Zeng Y., Huang T.Y., Xu H., Zhao Y. (2020). Molecular and Cellular Mechanisms Underlying the Pathogenesis of Alzheimer’s Disease. Mol. Neurodegener..

[3] Surguchov A. (2020). Caveolin: A New Link Between Diabetes and AD. Cell. Mol. Neurobiol..

[4] Vassilaki M., Aakre J.A., Kremers W.K., Mielke M.M., Geda Y.E., Alhurani R.E., Dutt T., Machulda M.M., Knopman D.S., Vemuri P. (2019). The Association of Multimorbidity With Preclinical AD Stages and SNAP in Cognitively Unimpaired Persons. J. Gerontol. Ser. A.

[5] Zhong L., Wang Z., Wang D., Wang Z., Martens Y.A., Wu L., Xu Y., Wang K., Li J., Huang R. (2018). Amyloid-Beta Modulates Microglial Responses by Binding to the Triggering Receptor Expressed on Myeloid Cells 2 (TREM2). Mol. Neurodegener..

[6] Wyssenbach A., Quintela T., Llavero F., Zugaza J.L., Matute C., Alberdi E. (2016). Amyloid β-Induced Astrogliosis Is Mediated by Β1-Integrin via NADPH Oxidase 2 in Alzheimer’s Disease. Aging Cell.

[7] Henstridge C.M., Hyman B.T., Spires-Jones T.L. (2019). Beyond the Neuron-Cellular Interactions Early in Alzheimer Disease Pathogenesis. Nat. Rev. Neurosci..

[8] Neumann H., Kotter M.R., Franklin R.J.M. (2009). Debris Clearance by Microglia: An Essential Link between Degeneration and Regeneration. Brain.

[9] Streit W.J., Khoshbouei H., Bechmann I. (2021). The Role of Microglia in Sporadic Alzheimer’s Disease. J. Alzheimer’s Dis..

[10] Gratuze M., Leyns C.E.G., Holtzman D.M. (2018). New Insights into the Role of TREM2 in Alzheimer’s Disease. Mol. Neurodegener..

[11] Brown G.C., St George-Hyslop P. (2022). Does Soluble TREM2 Protect Against Alzheimer’s Disease?. Front. Aging Neurosci..

[12] Yang J., Fu Z., Zhang X., Xiong M., Meng L., Zhang Z. (2020). TREM2 Ectodomain and Its Soluble Form in Alzheimer’s Disease. J. Neuroinflamm..

[13] Sofroniew M.V. (2020). Astrocyte Reactivity: Subtypes, States, and Functions in CNS Innate Immunity. Trends Immunol..

[14] Lines J., Baraibar A.M., Fang C., Martin E.D., Aguilar J., Lee M.K., Araque A., Kofuji P. (2022). Astrocyte-Neuronal Network Interplay Is Disrupted in Alzheimer’s Disease Mice. Glia.

[15] Elahi F.M., Casaletto K.B., La Joie R., Walters S.M., Harvey D., Wolf A., Edwards L., Rivera-Contreras W., Karydas A., Cobigo Y. (2020). Plasma Biomarkers of Astrocytic and Neuronal Dysfunction in Early- and Late-Onset Alzheimer’s Disease. Alzheimers. Dement..

[16] Morenas-Rodríguez E., Li Y., Nuscher B., Franzmeier N., Xiong C., Suárez-Calvet M., Fagan A.M., Schultz S., Gordon B.A., Benzinger T.L.S. (2022). Soluble TREM2 in CSF and Its Association with Other Biomarkers and Cognition in Autosomal-Dominant Alzheimer’s Disease: A Longitudinal Observational Study. Lancet Neurol..

[17] Suárez-Calvet M., Kleinberger G., Araque Caballero M.Á., Brendel M., Rominger A., Alcolea D., Fortea J., Lleó A., Blesa R., Gispert J.D. (2016). STREM2 Cerebrospinal Fluid Levels Are a Potential Biomarker for Microglia Activity in Early-Stage Alzheimer’s Disease and Associate with Neuronal Injury Markers. EMBO Mol. Med..

[18] Suárez-Calvet M., Morenas-Rodríguez E., Kleinberger G., Schlepckow K., Caballero M.Á.A., Franzmeier N., Capell A., Fellerer K., Nuscher B., Eren E. (2019). Early Increase of CSF STREM2 in Alzheimer’s Disease Is Associated with Tau Related-Neurodegeneration but Not with Amyloid-β Pathology. Mol. Neurodegener..

[19] Ewers M., Biechele G., Suárez-Calvet M., Sacher C., Blume T., Morenas-Rodriguez E., Deming Y., Piccio L., Cruchaga C., Kleinberger G. (2020). Higher CSF STREM2 and Microglia Activation Are Associated with Slower Rates of Beta-Amyloid Accumulation. EMBO Mol. Med..

[20] Sadick J.S., O’Dea M.R., Hasel P., Dykstra T., Faustin A., Liddelow S.A. (2022). Astrocytes and Oligodendrocytes Undergo Subtype-Specific Transcriptional Changes in Alzheimer’s Disease. Neuron.

[21] Perez-Nievas B.G., Serrano-Pozo A. (2018). Deciphering the Astrocyte Reaction in Alzheimer’s Disease. Front. Aging Neurosci..

[22] Van Hulle C., Jonaitis E.M., Betthauser T.J., Batrla R., Wild N., Kollmorgen G., Andreasson U., Okonkwo O., Bendlin B.B., Asthana S. (2021). An Examination of a Novel Multipanel of CSF Biomarkers in the Alzheimer’s Disease Clinical and Pathological Continuum. Alzheimer’s Dement..

[23] Benedet A.L., Milà-Alomà M., Vrillon A., Ashton N.J., Pascoal T.A., Lussier F., Karikari T.K., Hourregue C., Cognat E., Dumurgier J. (2021). Differences Between Plasma and Cerebrospinal Fluid Glial Fibrillary Acidic Protein Levels Across the Alzheimer Disease Continuum. JAMA Neurol..

[24] Cristóvaõ J.S., Gomes C.M. (2019). S100 Proteins in Alzheimer’s Disease. Front. Neurosci..

[25] Bellaver B., Ferrari-Souza J.P., Uglione da Ros L., Carter S.F., Rodriguez-Vieitez E., Nordberg A., Pellerin L., Rosa-Neto P., Leffa D.T., Zimmer E.R. (2021). Astrocyte Biomarkers in Alzheimer Disease: A Systematic Review and Meta-Analysis. Neurology.

[26] Zimmer E.R., Parent M.J., Souza D.G., Leuzy A., Lecrux C., Kim H.I., Gauthier S., Pellerin L., Hamel E., Rosa-Neto P. (2017). [18F]FDG PET Signal Is Driven by Astroglial Glutamate Transport. Nat. Neurosci..

[27] Rocha A., Bellaver B., Souza D.G., Schu G., Fontana I.C., Venturin G.T., Greggio S., Fontella F.U., Schiavenin M.L., Machado L.S. (2022). Clozapine Induces Astrocyte-Dependent FDG-PET Hypometabolism. Eur. J. Nucl. Med. Mol. Imaging.

[28] Pellerin L., Magistretti P.J. (2012). Sweet Sixteen for ANLS. J. Cereb. Blood Flow Metab..

[29] Pellerin L., Magistretti P.J. (1994). Glutamate Uptake into Astrocytes Stimulates Aerobic Glycolysis: A Mechanism Coupling Neuronal Activity to Glucose Utilization. Proc. Natl. Acad. Sci. USA.

[30] Xiang X., Wind K., Wiedemann T., Blume T., Shi Y., Briel N., Beyer L., Biechele G., Eckenweber F., Zatcepin A. (2021). Microglial Activation States Drive Glucose Uptake and FDG-PET Alterations in Neurodegenerative Diseases. Sci. Transl. Med..

[31] Salvadó G., Shekari M., Falcon C., Operto G.D.S., Milà-Alomà M., Sánchez-Benavides G., Cacciaglia R., Arenaza-Urquijo E., Niñerola-Baizán A., Perissinotti A.D.S. (2022). Brain Alterations in the Early Alzheimer’s Continuum with Amyloid-β, Tau, Glial and Neurodegeneration CSF Markers. Brain Commun..

[32] Salvadó G., Milà-Alomà M., Shekari M., Ashton N.J., Operto G., Falcon C., Cacciaglia R., Minguillon C., Fauria K., Niñerola-Baizán A. (2022). Reactive Astrogliosis Is Associated with Higher Cerebral Glucose Consumption in the Early Alzheimer’s Continuum. Eur. J. Nucl. Med. Mol. Imaging.

[33] Biel D., Suárez-Calvet M., Hager P., Rubinski A., Dewenter A., Steward A., Roemer S., Ewers M., Haass C., Brendel M. (2023). STREM2 Is Associated with Amyloid-Related p-Tau Increases and Glucose Hypermetabolism in Alzheimer’s Disease. EMBO Mol. Med..

[34] McKhann G.M., Knopman D.S., Chertkow H., Hyman B.T., Jack C.R., Kawas C.H., Klunk W.E., Koroshetz W.J., Manly J.J., Mayeux R. (2011). The Diagnosis of Dementia Due to Alzheimer’s Disease: Recommendations from the National Institute on Aging-Alzheimer’s Association Workgroups on Diagnostic Guidelines for Alzheimer’s Disease. Alzheimers. Dement..

[35] Jack C.R., Bennett D.A., Blennow K., Carrillo M.C., Dunn B., Haeberlein S.B., Holtzman D.M., Jagust W., Jessen F., Karlawish J. (2018). NIA-AA Research Framework: Toward a Biological Definition of Alzheimer’s Disease. Alzheimer’s Dement..

[36] Boellaard R., Delgado-Bolton R., Oyen W.J.G., Giammarile F., Tatsch K., Eschner W., Verzijlbergen F.J., Barrington S.F., Pike L.C., Weber W.A. (2015). FDG PET/CT: EANM Procedure Guidelines for Tumour Imaging: Version 2.0. Eur. J. Nucl. Med. Mol. Imaging.

[37] Chiaravalloti A., Barbagallo G., Martorana A., Castellano A.E., Ursini F., Schillaci O. (2019). Brain Metabolic Patterns in Patients with Suspected Non-Alzheimer’s Pathophysiology (SNAP) and Alzheimer’s Disease (AD): Is [18F] FDG a Specific Biomarker in These Patients?. Eur. J. Nucl. Med. Mol. Imaging.

[38] Marcus C., Mena E., Subramaniam R.M. (2014). Brain PET in the Diagnosis of Alzheimer’s Disease. Clin. Nucl. Med..

[39] D’Agostino E., Maes F., Vandermeulen D., Suetens P. (2007). Atlas-to-Image Non-Rigid Registration by Minimization of Conditional Local Entropy. Lect. Notes Comput. Sci. Incl. Subser. Lect. Notes Artif. Intell. Lect. Notes Bioinform..

[40] Mazziotta J., Toga A., Evans A., Fox P., Lancaster J., Zilles K., Woods R., Paus T., Simpson G., Pike B. (2001). A Four-Dimensional Probabilistic Atlas of the Human Brain. J. Am. Med. Inform. Assoc..

[41] Mazziotta J.C., Toga A.W., Evans A., Fox P., Lancaster J. (1995). A Probabilistic Atlas of the Human Brain: Theory and Rationale for Its Development. Neuroimage.

[42] Bennett C.M., Wolford G.L., Miller M.B. (2009). The Principled Control of False Positives in Neuroimaging. Soc. Cogn. Affect. Neurosci..

[43] Pagani M., De Carli F., Morbelli S., Öberg J., Chincarini A., Frisoni G.B., Galluzzi S., Perneczky R., Drzezga A., Van Berckel B.N.M. (2015). Volume of Interest-Based [18F]Fluorodeoxyglucose PET Discriminates MCI Converting to Alzheimer’s Disease from Healthy Controls. A European Alzheimer’s Disease Consortium (EADC) Study. NeuroImage Clin..

[44] An Y., Varma V.R., Varma S., Casanova R., Dammer E., Pletnikova O., Chia C.W., Egan J.M., Ferrucci L., Troncoso J. (2018). Evidence for Brain Glucose Dysregulation in Alzheimer’s Disease. Alzheimers Dement..

[45] Soto-Rojas L.O., Pacheco-Herrero M., Martínez-Gómez P.A., Campa-Córdoba B.B., Apátiga-Pérez R., Villegas-Rojas M.M., Harrington C.R., de la Cruz F., Garcés-Ramírez L., Luna-Muñoz J. (2021). The Neurovascular Unit Dysfunction in Alzheimer’s Disease. Int. J. Mol. Sci..

[46] Semprini R., Koch G., Belli L., Lorenzo F.D., Ragonese M., Manenti G., Sorice G.P., Martorana A. (2016). Insulin and the Future Treatment of Alzheimer’s Disease. CNS Neurol. Disord. Drug Targets.

[47] Bargers S.W., Van Eldikso1 L.J. (1992). S100B Stimulates Calcium Fluxes in Glial and Neuronal Cells. J. Biol. Chem..

[48] Nishiyama H., Knöpfel T., Endo S., Itohara S. (2002). Glial Protein S100B Modulates Long-Term Neuronal Synaptic Plasticity. Proc. Natl. Acad. Sci. USA.

[49] Winocur G., Roder J., Lobaugh N. (2001). Learning and Memory in S100-Beta Transgenic Mice: An Analysis of Impaired and Preserved Function. Neurobiol. Learn. Mem..

[50] Busche M.A., Konnerth A. (2016). Impairments of Neural Circuit Function in Alzheimer’s Disease. Philos. Trans. R. Soc. B Biol. Sci..

[51] Maestú F., de Haan W., Busche M.A., DeFelipe J. (2021). Neuronal Excitation/Inhibition Imbalance: Core Element of a Translational Perspective on Alzheimer Pathophysiology. Ageing Res. Rev..

[52] Ambrad Giovannetti E., Fuhrmann M. (2019). Unsupervised Excitation: GABAergic Dysfunctions in Alzheimer’s Disease. Brain Res..

[53] Zott B., Simon M.M., Hong W., Unger F., Chen-Engerer H.J., Frosch M.P., Sakmann B., Walsh D.M., Konnerth A. (2019). A Vicious Cycle of β Amyloid−dependent Neuronal Hyperactivation. Science.

[54] Brezovakova V., Sykova E., Jadhav S. (2022). Astrocytes Derived from Familial and Sporadic Alzheimer’s Disease IPSCs Show Altered Calcium Signaling and Respond Differently to Misfolded Protein Tau. Cells.

[55] Friederici A.D. (2011). The Brain Basis of Language Processing: From Structure to Function. Physiol. Rev..

[56] Fogassi L., Ferrari P.F., Gesierich B., Rozzi S., Chersi F., Rizzolotti G. (2005). Neuroscience: Parietal Lobe: From Action Organization to Intention Understanding. Science.

[57] Giannakopoulos P., Kövari E., Herrmann F.R., Hof P.R., Bouras C. (2009). Interhemispheric Distribution of Alzheimer Disease and Vascular Pathology in Brain Aging. Stroke.

[58] Chiaravalloti A., Barbagallo G., Ricci M., Martorana A., Ursini F., Sannino P., Karalis G., Schillaci O. (2018). Brain Metabolic Correlates of CSF Tau Protein in a Large Cohort of Alzheimer’s Disease Patients: A CSF and FDG PET Study. Brain Res..

